# Supplementary material for: Telomeric repeat-containing RNA increases in aged human cells
Source: Nucleic Acids Res. 2025 Jul 10;53(13):gkaf597. doi: 10.1093/nar/gkaf597 (PMC12242772; doi:10.1093/nar/gkaf597)
Supplement: gkaf597_Supplemental_Files [file gkaf597_supplemental_files.zip › Supp_info_NAR_Hsieh_June_2025.pdf]

Supplementary Information

Telomeric Repeat-Containing RNA Increases in Aged Human Cells

Yu-Hung Hsieh[1,#], Chin-Hua Tai,[1,#], Meng-Ting Yeh[1,#], Yu-Chen Chen[1], Po-Cheng Yang[1], Chien-Ping Yen[1], Hong-Jhih Shen[1], Chan-Hsien Yeh[2,3], Hung-Chih Kuo[2,3], Der-Sheng Han[4,5,]* and Hsueh-Ping Catherine Chu[1,]*

[1] Institute of Molecular and Cellular Biology, National Taiwan University, No. 1 Sec. 4 Roosevelt Road, Taipei, Taiwan.

[2]Graduate Institute of Medical Genomics and Proteomics, National Taiwan University College of Medicine, Taipei, 10051, Taiwan

[3]Institute of Cellular and Organismic Biology, Academia Sinica, Taipei 11529, Taiwan

[4]Department of Physical Medicine and Rehabilitation, National Taiwan University Hospital, Bei-Hu Branch, Taipei 100, Taiwan.

[5]Department of Physical Medicine and Rehabilitation, College of Medicine, National Taiwan University.

[#]These authors contributed equally

* To whom correspondence should be addressed. Email: cchu2017@ntu.edu.tw (Hsueh-Ping Catherine Chu), dshan1121@yahoo.com.tw (Der-Sheng Han)

This supplementary information includes 9 Supplementary Figures.

A

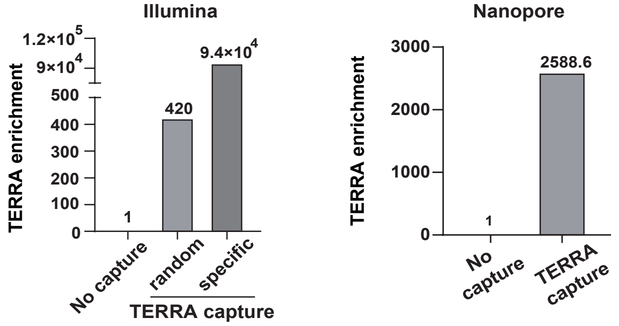

B

TERRA transcription regions

Types	chromosome end	Schematic diagram
type I	chr1p, chr1q, chr2p, chr2q, chr3p, chr3q, chr4p, chr4q, chr5p, chr5q, chr6p, chr6q, chr7p, chr7q, chr8p, chr9p, chr9q, chr10q, chr11p, chr11q, chr12p, chr13q, chr14q, chr15p, chr15q, chr16p, chr16q, chr17q, chr18p, chr18q, chr19p, chr19q, chr20p, chr20p-2, chr20q, chr21q, chr22q, chrXq	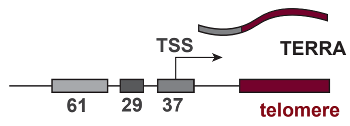
type II	chr10p chr17p chr5p-2 chr18p-2	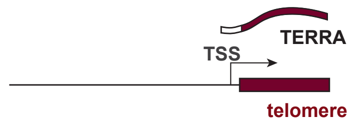
type III	· TERRA transcribed from interstitial telomeric repeats. (19 sites)	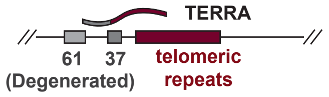
Not detected	chr8q, chr12q, chr13p, chr14p, chr21p, chr22p, chrXp	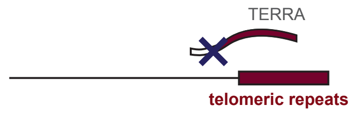

C

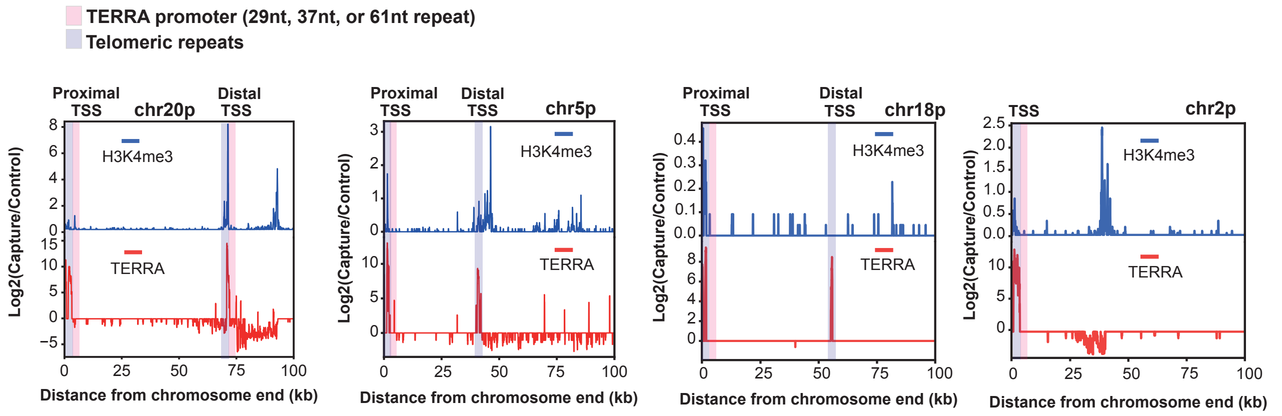

Supplementary Figure S1. Identification of TERRA transcription regions by short-read and long-read RNA-sequencing in human cells. (**A**) RT-qPCR results show TERRA enrichment in U2OS cells after TERRA capture for Illumina RNA-seq and Nanopore direct-RNA sequencing. cDNA synthesis was performed using either random or telomeric-specific primers for Illumina RNA-seq. (**B**) Summary of different types of TERRA transcription regions with or without 61-29-37 bp repeats at TERRA promoters. TERRA transcribed from chromosome ends (Type I and Type II). TERRA transcribed from interstitial telomeric repeats (Type III). TERRA enrichment and Nanopore reads were not detected in some chromosome ends. (**C**) TERRA enrichment and H3K4me3 coverage are shown at indicated chromosome ends (100 kb from telomeric repeat tracts). Two transcription start sites (TSS) of TERRA were identified at the ends of chr20p, chr5p, and chrp18p. One TSS of TERRA was identified at most chromosome ends, such as chr2p.

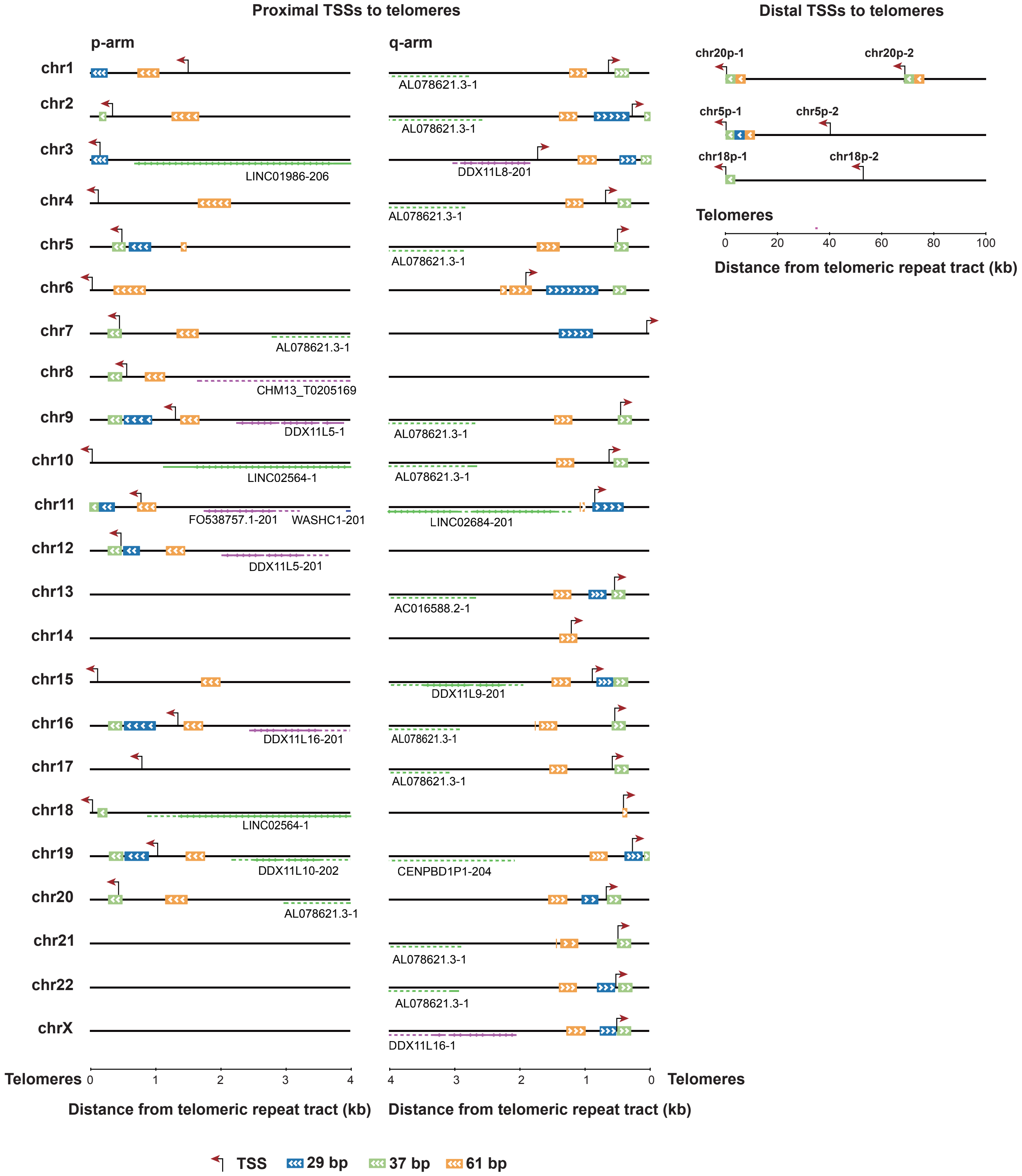

Supplementary Figure S2. Illustration of the distance of the identified TERRA transcription start sites from their telomeric ends. The distance of TERRA transcription start sites (TSSs) to the telomeric repeat tracts (position 0) are shown. TERRA promoters with 29-37-61 bp repeats are indicated for each chromsome end. 39 proximal transcription start sites (TSSs) for TERRA were identified near chromosome ends (left), alone with 3 additional distal candidate TSSs (chr20p-2, chr5p-2 and chr18p-2) located further from telomeres (right).

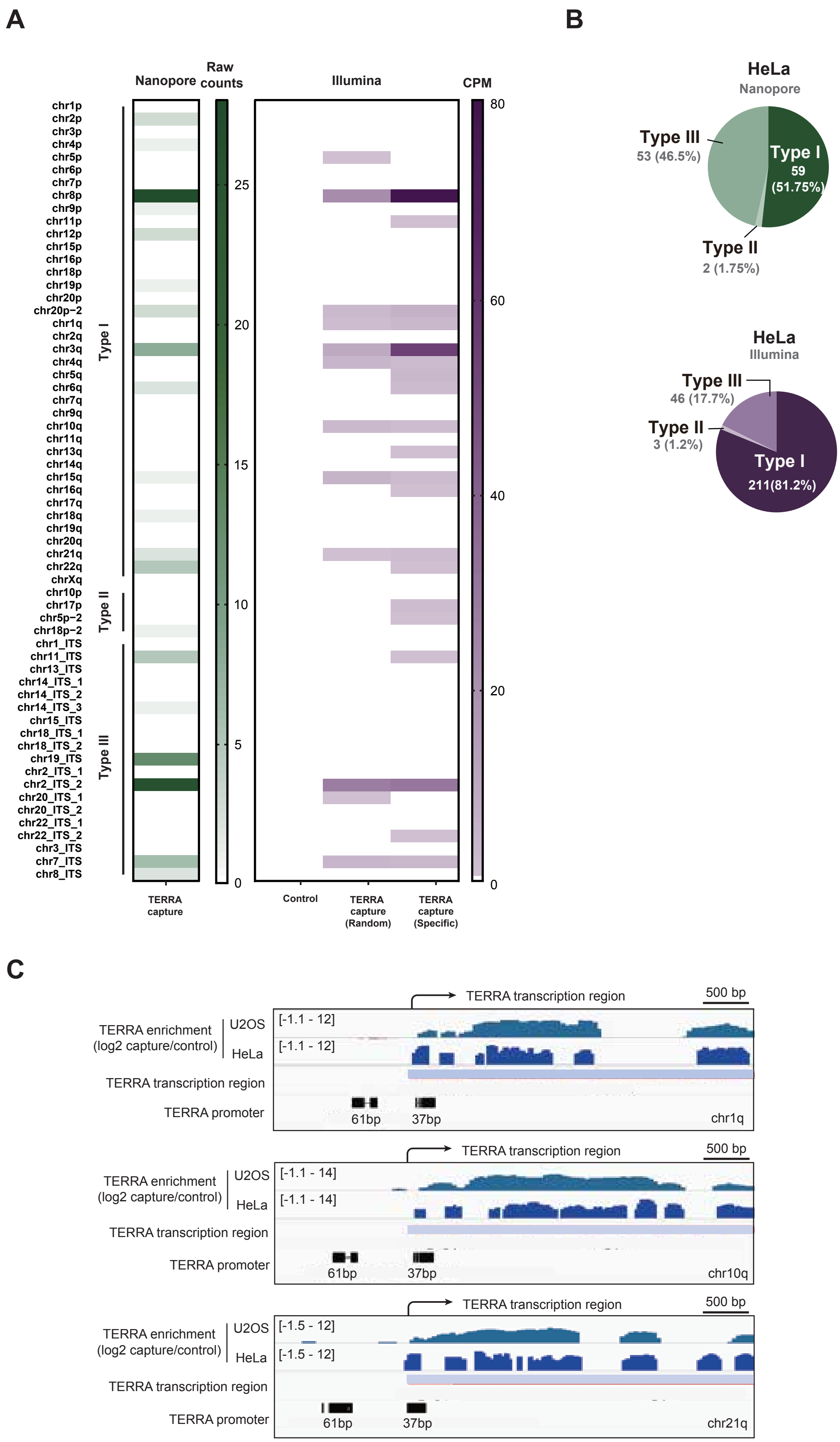

Supplementary Figure S3. TERRA-capture RNA-seq in HeLa cells
TERRA-captured RNA-seq was conducted using HeLa cells to assess TERRA enrichment in the TERRA transcription regions. (**A**) Heatmap showing TERRA counts from Nanopore long reads or illumina sequencing. (**B**) Pie charts show the numbers of Type I, II, III TERRA reads from Nanopore or Illumina sequencning in HeLa cells. (**C**) Genome browser screenshots depicting TERRA enrichment (specifc groups) in U2OS and HeLa cells, showing a similar pattern.

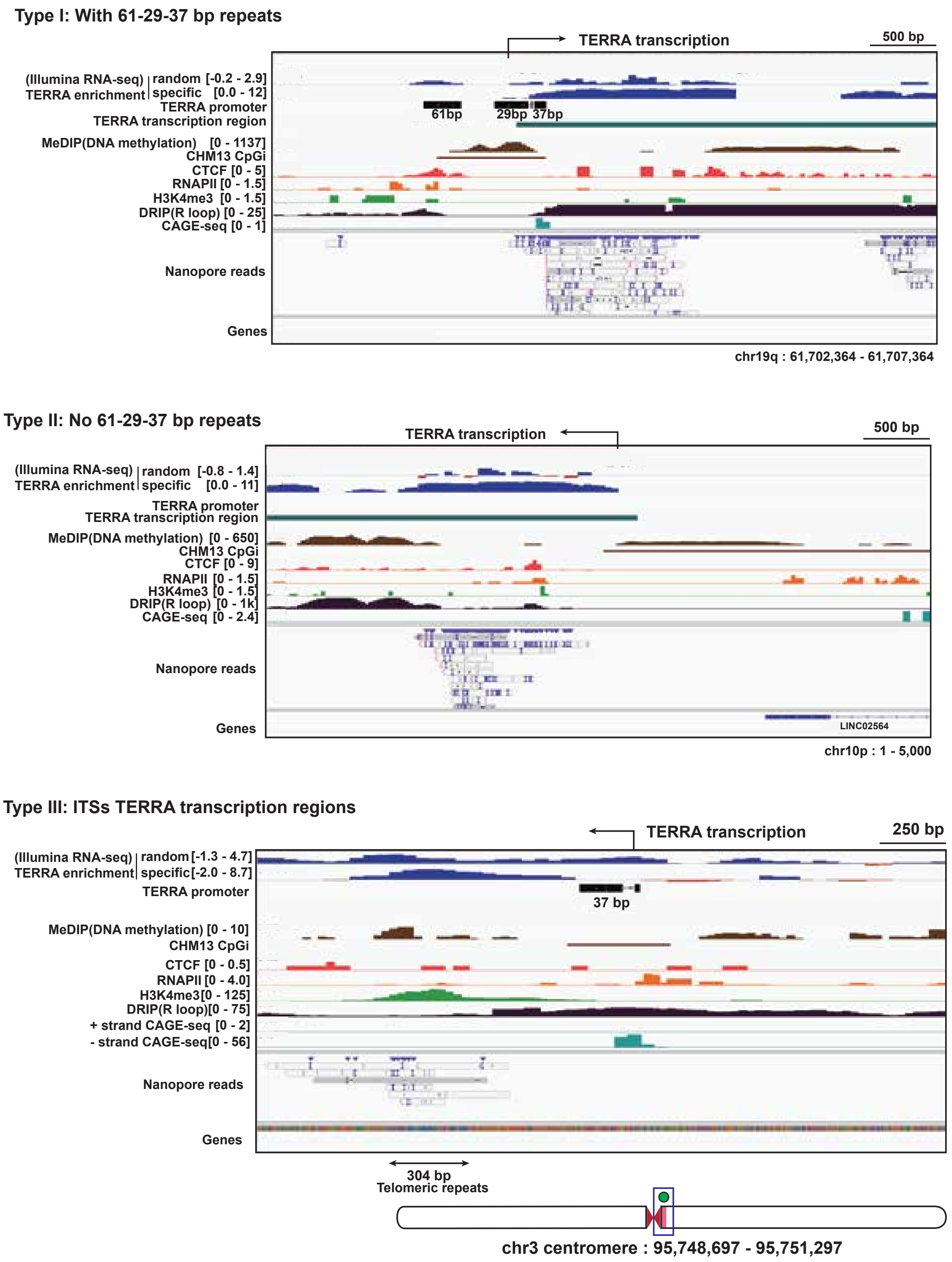

Supplementary Figure S4. Genome browser view of different types of TERRA transcription regions.
Example of Type I TERRA transcription regions on chr19q arm with the 61-29-37 bp repeat promoter. Example of Type II TERRA transcription regions on chr10p arm without the 61-29-37 bp repeat promoter. Example of Type III TERRA transcription regions at interstitial telomeric repeats on chr3 near centromere. The coverages of epigenetic marks such as DNA methylation (MeDIP), H3K4me3, R-loops (DRIP-seq), CTCF, and RNA Pol II are shown near TERRA transcription start sites. All sequecing data were obtained from U2OS cells, except for CAGE-seq, which were obtained from HeLa cells.

A

Class	61 bp	29 bp	37 bp	chr end	summary
+ 29bp	O	O	O	16	19
	X	O	O	0	
	O	O	X	1	
	X	O	X	2	
- 29bp	O	X	O	12	18
	O	X	X	5	
	X	X	O	1	

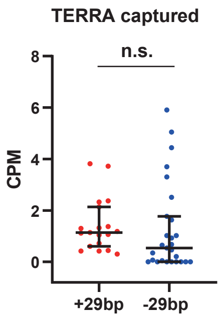

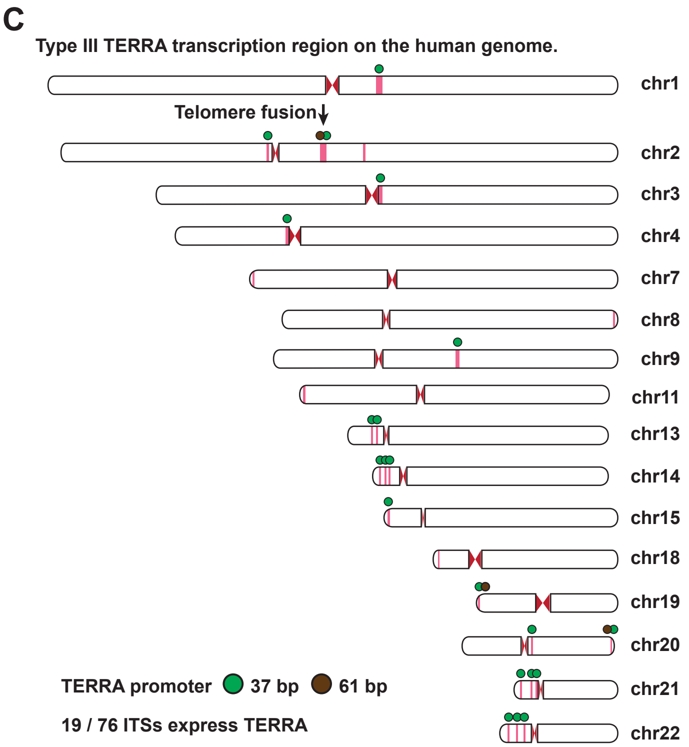

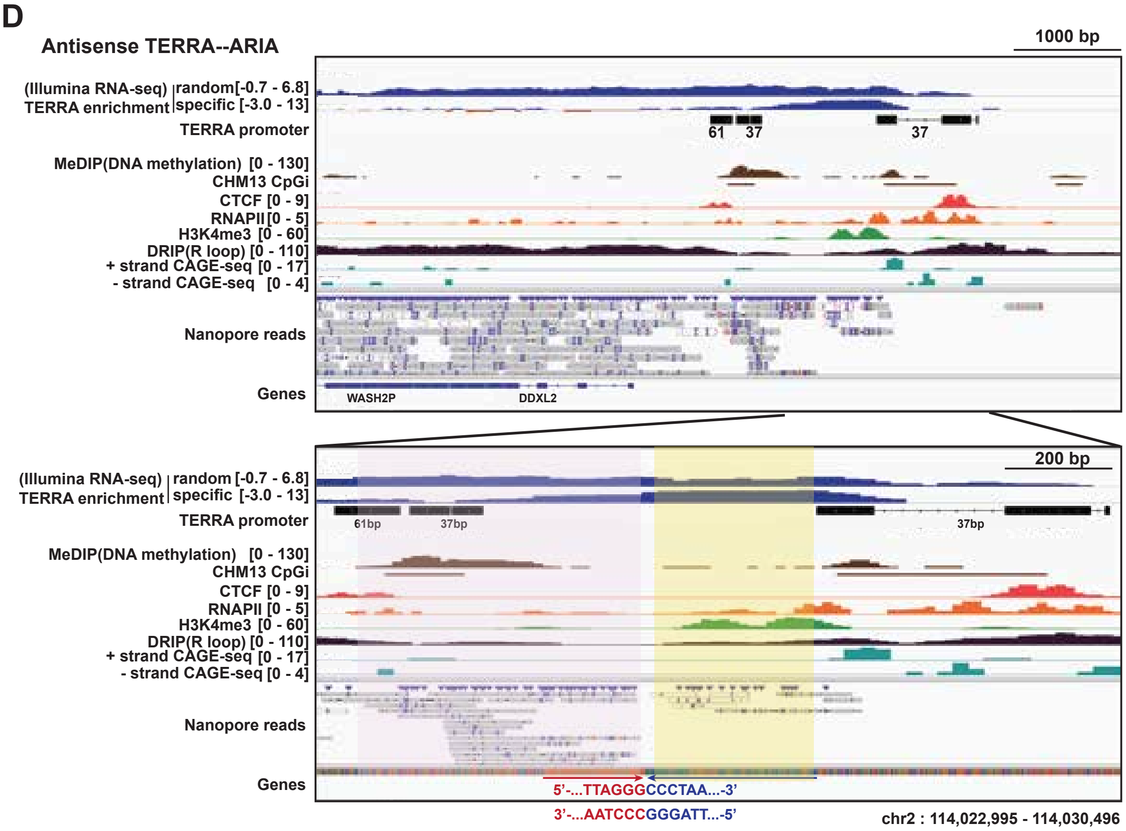

Supplementary Figure S5. TERRA arising from interstitial telomeric sequences
(**A**) The table provides the numbers of the combination of 61-29-37 bp repeats at chromosome ends of Type I TERRA transcription regions. The presence (O) or absence (X) of the 61, 29, 37 bp repetitive sequences. (**B**) Dot plots showing the TERRA levels (CPM, counts per million) at individual chromosome ends with or without the 29 bp repeat element in Type I TERRA transcription regions in U2OS cells. n.s. no significance, by Mann-Whitney U test. (**C**) Schematic illustration of Type III TERRA transcription regions. The pink strips indicate the locations of interstitial telomeric repeat sequences (ITSs) that generate TERRA. Only the regions exhibiting TERRA enrichment and Nanopore reads are shown. The 61-37 bp repeat promoters are represented by brown and green dots. Notably, no 29 bp repeats were identified near the TERRA transcription regions. The pie chart displays the ratio of Type III TERRA transcription regions with or without 61-37 bp repeat promoters among the ITSs regions that contain telomeric repeats longer than 200 nt. (**D**) ARIA transcripts with C-rich telomeric sequences were aligned to a region containing a telomere-telomere fusion site on Chr2 (light pink shade) in U2OS cells. TERRA transcripts from Nanopore reads with G-rich telomeric sequences are also detected near the fusion site (yellow shade).

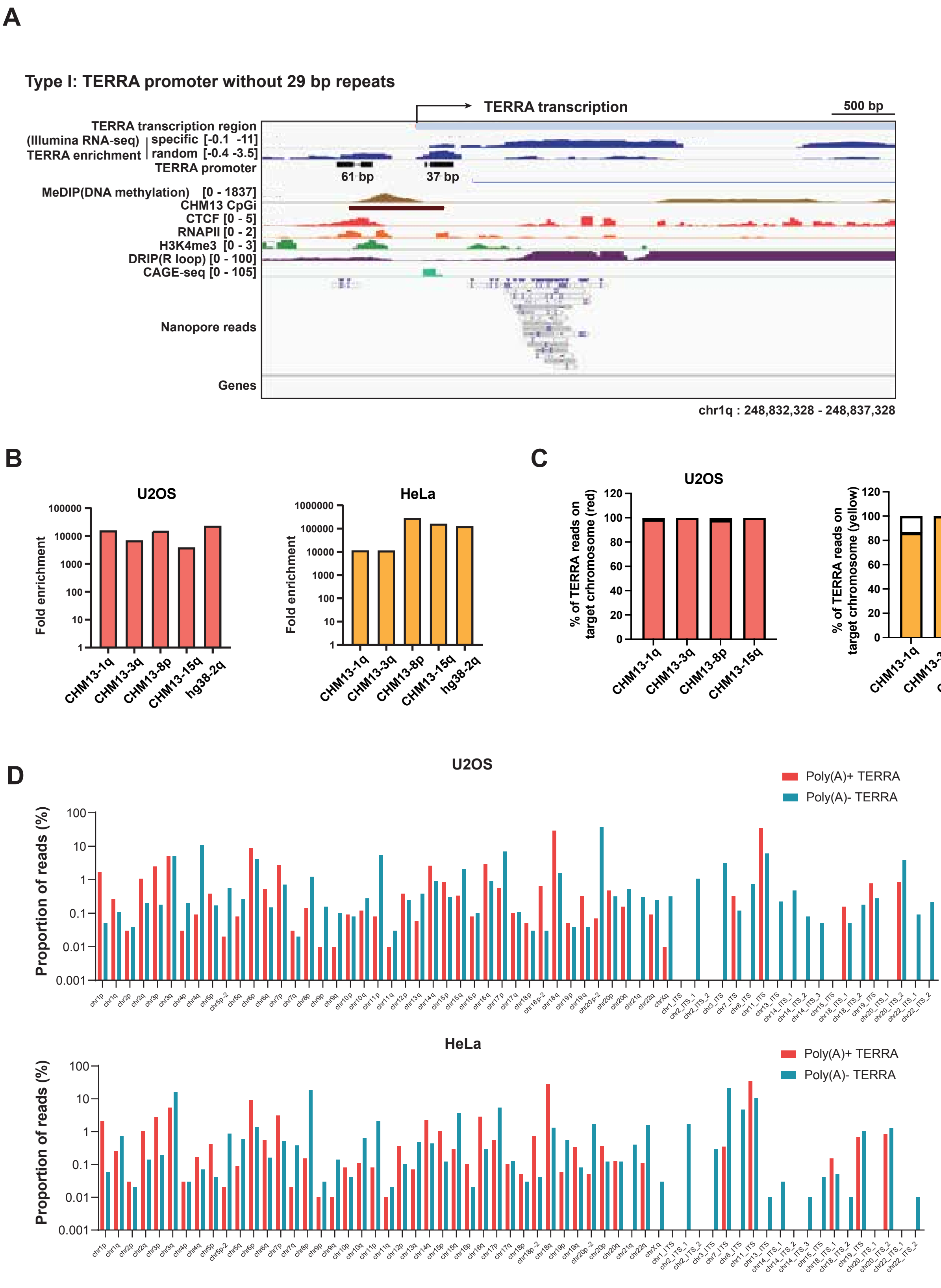

Supplementary Figure S6. Telomeric length in TERRA transcription regions.

(**A**) Type I TERRA transcription region containing the 61-37 bp repeat promoter without canonical 29 bp repeats. Methylation on CpG islands is present in the region between 61 bp and 37 bp repeats. All sequencing data were from U2OS cells, except for CAGE-seq (from HeLa cells).(**B**) RT-qPCR analysis to assess TERRA enrichment using CHM13 subtelomeric primers following TERRA capture in U2OS and HeLa cells. Fold enrichment was calculated by TERRA capture v.s. no-capture. (**C**) NGS sequencing for TERRA RT-qPCR products. Subtelomeric primers were designed according to the T2T CHM13 reference genome. TERRA reads mapped to target chromosome ends over all subtelomeric reads in TERRA transcription regions, depicted as "% of TERRA reads on target chromosome" in colors. (**D**) Percentage of total TERRA reads in poly(A)+ and poly(A)- fractions for each TERRA transcription region was shown in U2OS and HeLa cells.

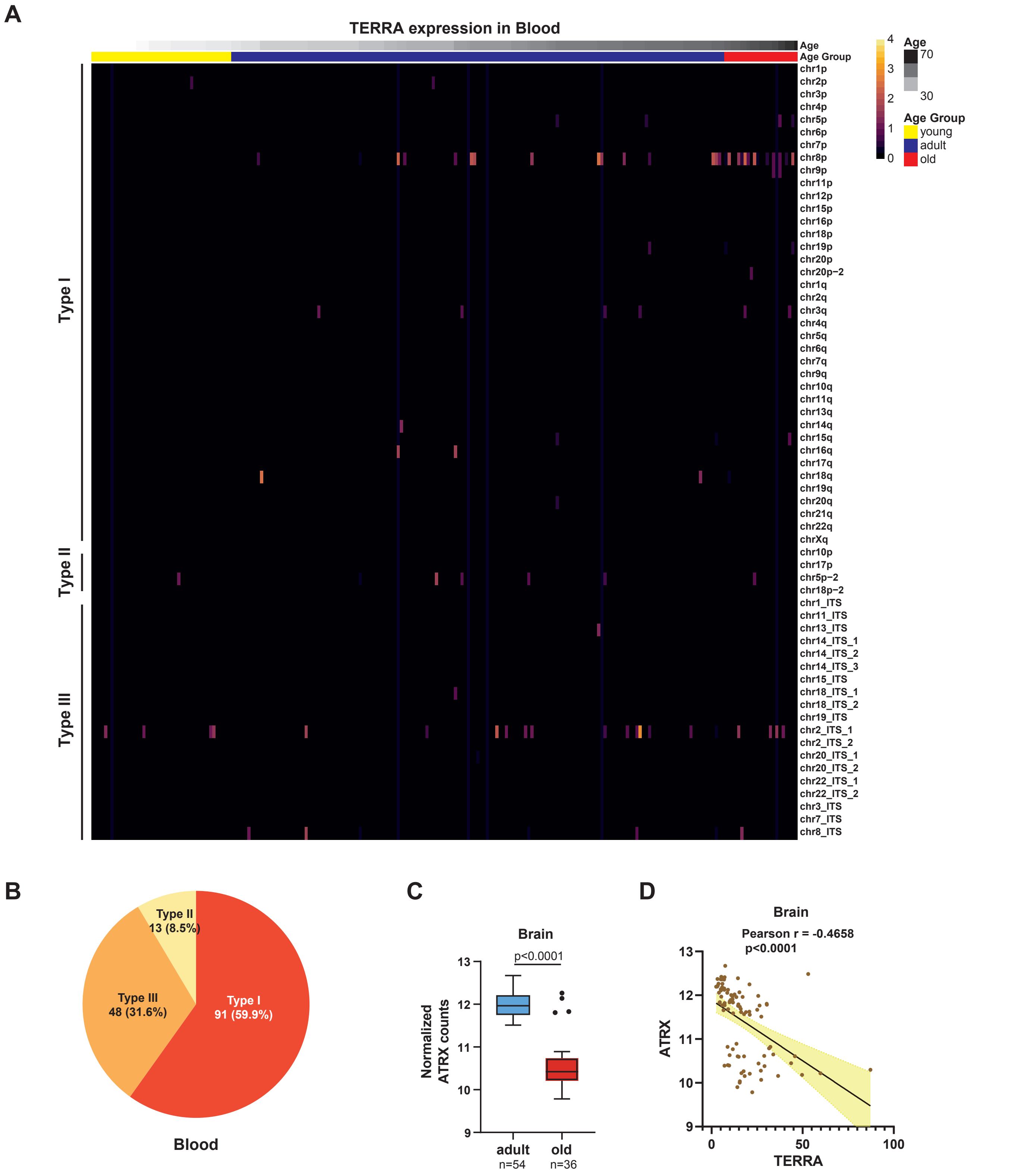

Supplementary Figure S7. TERRA expression increases with age in blood cells.
(**A**) Heatmap of normalized TERRA counts from different chromosome ends and ITSs in blood cells obtained from individuals across different ages. (**B**) Pie Chart showing Type I, II, III TERRA expression in blood cells. (**C**) Boxplots of normalized ATRX counts in brain tissues. Bars, median with interquartile. P values, by Mann-Whitney U test. (**D**) Scatter plots of normalized TERRA and ATRX counts in brain tissues. P values, by Pearson's correlation.

A

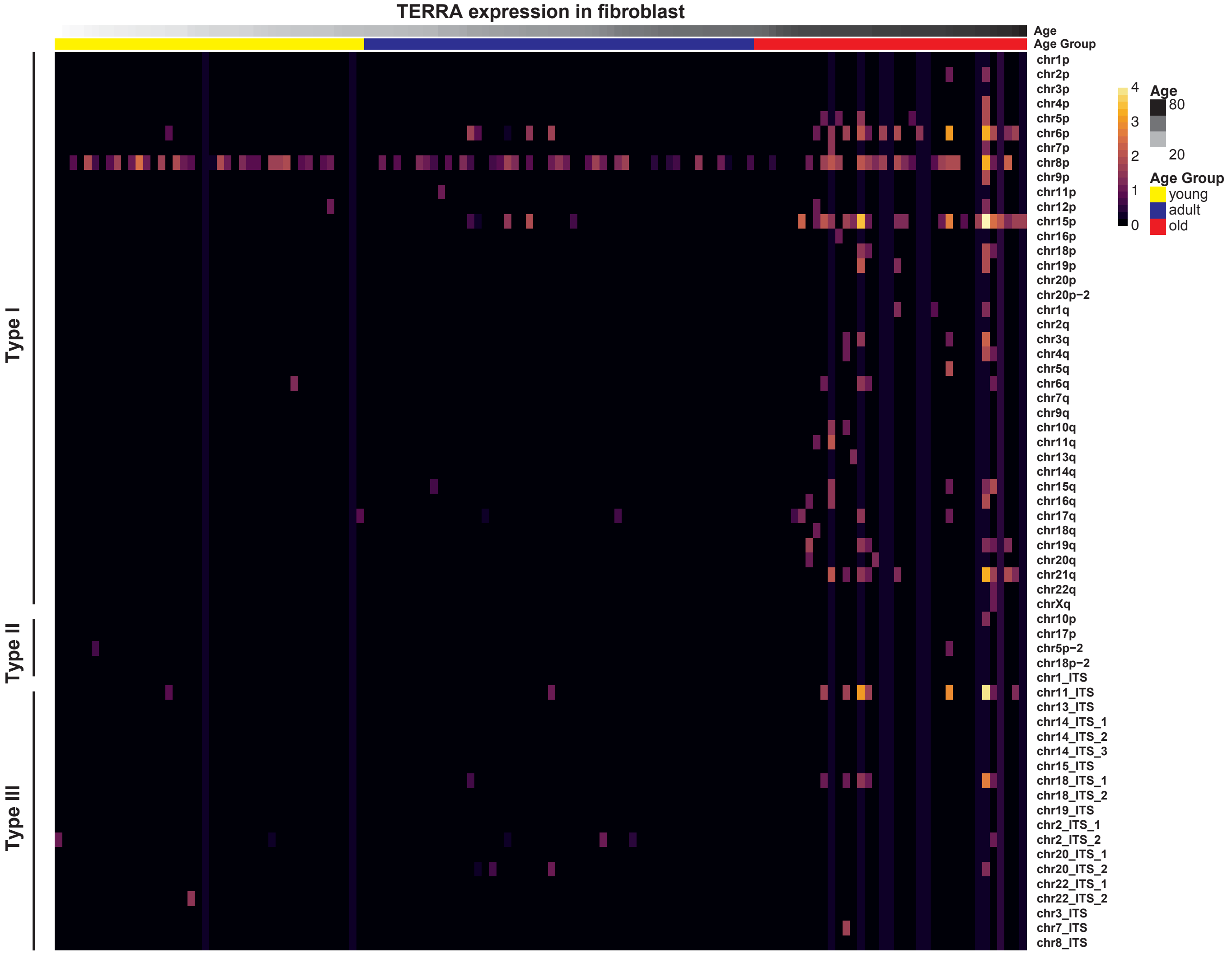

B

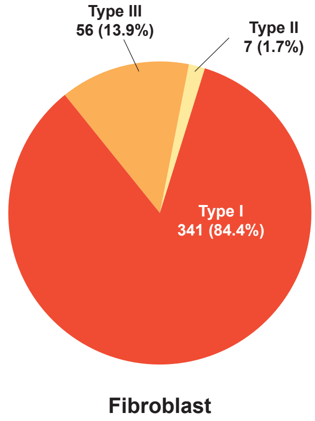

Supplementary Figure S8. TERRA expression increases with age in fibroblasts.
(**A**) Heatmap of normalized TERRA counts from different chromosome ends and ITSs in fibroblasts obtained from individuals across different ages.
(**B**) Pie Chart showingType I, II, III TERRA expression in fibroblasts.

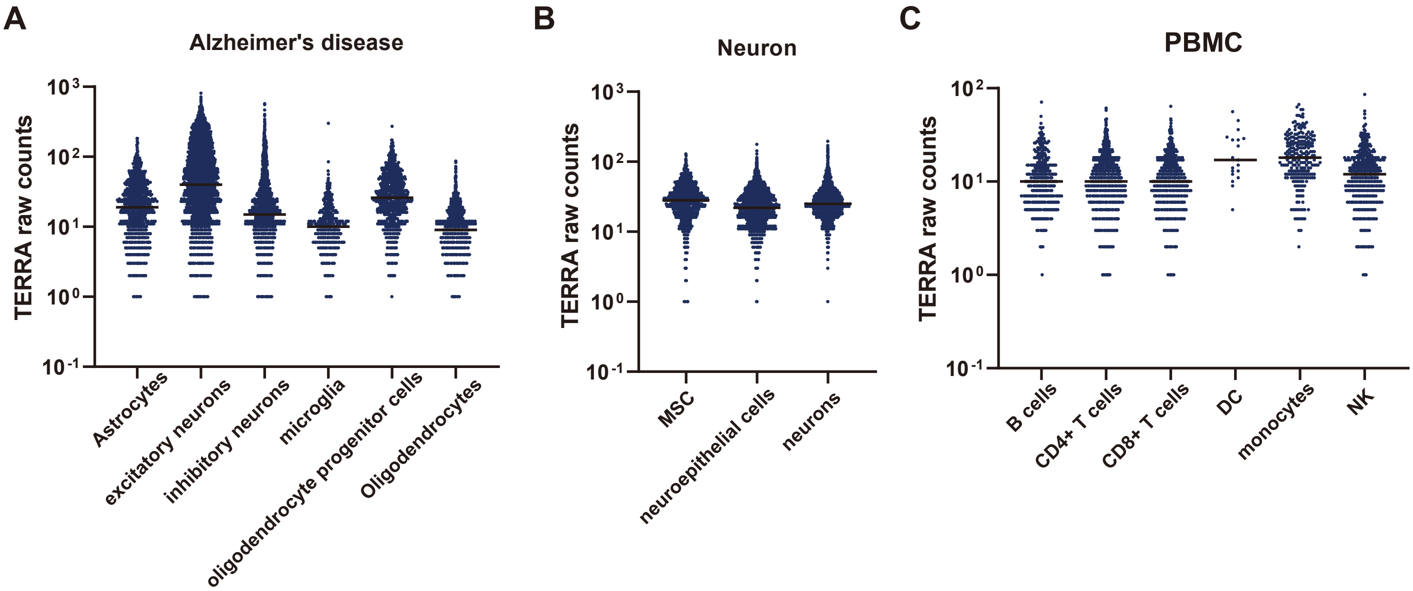

D

This study	Rodrigues, J., 2024	chromosome end	summary
o	o	chr1q,chr2p, chr3q, chr4q, chr5p, chr5q chr6q, chr7p, chr7q, chr8p, chr8q, chr9q, chr10p, chr10q, chr11p, chr11q,chr12p, chr13q, chr15q, chr16p,chr16q, chr17p, chr18p, chr18q,chr19p, chr20p, chr20q, chr21q, chr22q, chrXq	31
o	X	chr1p, chr2q, chr3p, chr4p, chr6p, chr14p, chr15p, chr19q	8
X	o	chr9p, chr12q, chr22p, chrXp	4
X	X	chr13p, chr14q, chr21p	3

Supplementary Figure S9. TERRA raw read counts in various tissues
(**A**) TERRA raw read counts per cell without YARN normalization. Single-nucleus RNA-seq for cells isolated from the prefrontal cortex of individuals with Alzheimer's disease. Each dot represents one single cell. Solid lines, median. (**B**) TERRA raw read counts per cell without YARN normalization. Single-cell RNA-seq analysis in human embryonic stem cells (hESCs) undergoing neuronal differentiation with various HOX patterning periods. MSC, mesenchymal stem cells. Each dot represents one single cell. Solid lines, median. (**C**) TERRA raw read counts per cell without YARN normalization. Single-cell RNA-seq analysis in PBMC. Each dot represents one single cell. Solid lines, median. (**D**) Comparison of detectable TERRA reads at chromosome ends between this study and the study by Rodrigues et al., 2024.
